# Commentary: The Experience of Depression during the Careers of Elite Male Athletes

**DOI:** 10.3389/fpsyg.2016.01869

**Published:** 2016-12-01

**Authors:** Alan Ringland

**Affiliations:** Health and Leisure, Institute of TechnologyTralee, Ireland

**Keywords:** mental health, depression, sport, multi-faceted, definition

Currently little is known about the prevalence and etiology of depression in the athletic population (Nixdorf et al., 2015). Almost 75% of mental health difficulties first emerge between the ages of 15 and 25 (Kessler et al., 2005). And arguably the peak years for elite sport performance overlap with the period where the risk of mental health disorders are highest. Given the context of sport and the nature of competition and youth sport, how many coaches, parents and sporting organizations have ever considered this evidence or acted upon it? Sport and mental health have been artificially decoupled due in part to mental health stigma.

Mental health stigma is a hurdle that is prevalent in the sport context and one that is particularly challenging in the patriarchal culture of elite sport. This paper draws attention to the poignant issue that men's experience of gender role conflict may be associated with an increased endorsement of stigmatization around mental health concerns (Gulliver et al., 2012). The consequences of mental health stigma include a decreased willingness to refer friends and family members experiencing a mental health concern to access relevant services. Social supports in the form of peers, teammates, coaches, sporting organizations and managers may paradoxically be perceived as linked to stressors. It is plausible that individuals and the organizational culture may be perceived by the individual as putting performance and achieving podium positions ahead of the athlete's well-being. As sporting performance is a key indicator of sporting excellence this may mask individual mental health issues. Arguably, the primacy of performance over the person is a challenge within elite sport. Admitting to a problem is viewed as acknowledging a weakness and this does not fit with the script of the “mentally tough athlete” as the authors propose in their interpretation of an “unhealthy” and “dysfunctional” relationship with sport.

As noted by Doherty et al. (2016), public perception tends to hold elite performers in superstar recognition as heroes rather than role models. Success and celebrity unfortunately offer little refuge into the wide area of mental health as recent biographies (e.g., Jonny Wilkinson, Victoria Pendleton) have illustrated (Newman et al., 2016). Three key questions arise. Firstly, is it beneficial for athletes to come out (i.e., public about their episodes) about their experience of depression? Secondly, to what extent does this act of public disclosure encourage other athletes to seek appropriate help? And finally, does this really change the nature and prevalence of mental health stigma in sport? It is possible that cognitive dissonance and social cognitive biases may increase service aversion (e.g., my symptoms aren't like their, I'm not under the pressures they were). Future research should explore the consequences for mental health stigma of public disclosures of depression by elite athletes on their peers, on their support staff (e.g., coaches) and on the public at large.

Doherty et al. (2016) do not provide comprehensive details (e.g., exclusion criteria; DSM V scores) beyond the transcripts on the thoughts, feelings, behaviors and physical symptoms (e.g., sleep and diet changes) of the elite male sporting athletes. The authors could have augmented their analysis by analyzing the nature of the language used by the athletes. The article has produced meaningful data but further exploration of the specific how's and whys of the causes, symptoms and coping mechanisms and the layers and levels of each category of depression deserves further attention. There are few examples of athletes using sport as a coping strategy and this may be explained by their lack of understanding of their self-regulatory strategies. What is striking is the masking of the depression symptoms outside of their sporting environment and their isolation from social support. Nevertheless, there are significant recommendations to be gleamed from this research for sporting bodies, coaches and support networks when dealing with individuals who behave, feel and think like the individual narratives conveyed in this research.

Experiencing mental health problems may be described as clinical or sub-clinical, for example. If we truly view mental health as a continuum, then the quality of an individual's mental health needs may provide insight into the possible levels and layers of the construct. Depression is fraught with inherent operational definitional difficulties because of the multi-layered and multifaceted nature which requires additional research from the clinical domain but also the different contextual environments. These may additionally highlight when and how individuals withdraw from their work, how and why conflicts develop and indicate the pattern of avoidance of seeking help. The suppression of emotion may have been misinterpreted in previous literature as mood states and depression have become interchangeably linked (Lane and Terry, 2000). An operational definition of depression as a construct is essential. While the author's acknowledge this limitation of depression it is an aspect worthy of further exploration.

Arguably, there needs to be care with the interpretation of some of the findings. Many of the symptoms of depression experienced by the male athletes in this study have been negatively portrayed and it is not known, as the data is retrospective, if the individual's perception was distorted by decaying memories. It was not always clear of the timelines of the stressors they faced in relation to their depressive episodes. Triangulation of the data by interviewing others within their sporting system or members of their social support structure would have provided a more valid and accurate narrative to emerge.

Athletes, particularly those recovering from injury, should be supported by all members of multi-disciplinary teams not just to seek help for mental disorders, but to develop their positive mental health through access to appropriate medical and sport science professionals. As a result, depression should be viewed as an inclusive challenge for all in high performance sport, from performers to practitioners. If we are to diminish the impact of mental health stigma then mental health is not simply an issue for an individual and their psychologist, but it is a challenge for sporting communities at large.

## Author contributions

The author confirms being the sole contributor of this work and approved it for publication.

### Conflict of interest statement

The author declares that the research was conducted in the absence of any commercial or financial relationships that could be construed as a potential conflict of interest.
